# Therapeutic Potential of *Acalypha wilkesiana* in Type 2 Diabetes: A Review

**DOI:** 10.1155/jdr/6278075

**Published:** 2025-11-13

**Authors:** Samuel Inshutiyimana, Patel Dineshbhai Yesh, Michael Matiop Aleu, Aibekhanya Nkosana Sebata, Kenfa Ajumesi, Norhayati

**Affiliations:** ^1^Department of Pharmaceutics and Pharmacy Practice, School of Pharmacy and Health Sciences, United States International University-Africa, Nairobi, Kenya; ^2^Department of Pharmaceutical Chemistry, Pharmacology and Pharmacognosy, School of Pharmacy and Health Sciences, United States International University-Africa, Nairobi, Kenya; ^3^Department of Pharmacy, Faculty of Pharmacy, Universitas Borneo Lestari, Banjarbaru, Indonesia

**Keywords:** *Acalypha wilkesiana*, antidiabetic, copper leaf, insulin resistance, phytochemicals, Type 2 diabetes

## Abstract

**Background:**

Type 2 diabetes mellitus (T2DM) refers to a chronic metabolic disorder that results from insulin resistance, leading to impaired insulin action and uncontrolled plasma glucose levels. *Acalypha wilkesiana* is among medicinal plants that are ethnobotanically used in the management of T2DM. However, there is a paucity of information on its antidiabetic potential. This review is aimed at providing a current understanding of the mechanism of action, potency, and safety of *Acalypha wilkesiana* in T2DM therapy.

**Methods:**

A narrative review was thoroughly conducted by searching Google Scholar and PubMed databases using a predefined combination of keywords. All gathered articles were reviewed for the content regarding T2DM, *Acalypha wilkesiana*, mechanism of action, and safety. A total of 44 articles were considered in this review.

**Results:**

Several experimental studies revealed that extracts of *Acalypha wilkesiana* inhibit *α*-glucosidase and *α*-amylase, which normally break down carbohydrates postprandially. Notably, the ethanolic root bark extracts of *Acalypha wilkesiana* have shown lower inhibitory concentrations compared to those of both the plant extracts in other solvents and the acarbose drug, emphasizing its greater potency. Additionally, the leaves of *Acalypha wilkesiana* have been reported to have no harmful effects on the red blood cells of diabetic rabbits and can even restore the alloxan-induced impairment of pancreas and spleen cells.

**Conclusion:**

*Acalypha wilkesiana* demonstrates antihyperglycemic activity and can reverse the dysfunction of critical organs. It promises advances in the development of antihyperglycemic agents which are more efficacious and safer than synthetic agents. However, clinical trials should be conducted to establish human-tailored doses, ensuring an improved safety profile of the plant in the treatment of T2DM.

## 1. Introduction

Type 2 diabetes mellitus (T2DM) is a chronic metabolic disorder characterized by insulin resistance, culminating in impaired insulin secretion and action. Consequently, glucose molecules accumulate in the blood vessels, leading to hyperglycemia and complications such as hyperosmolarity hyperglycemia state [1, 2]. In addition to insulin resistance, progressive dysfunction of pancreatic *β*-cells, increased hepatic glucose production, and low-grade inflammation are recognized contributors to the pathogenesis of T2DM. These mechanisms together accelerate the metabolic imbalance that underlies chronic hyperglycemia [3]. Although the disorder can be asymptomatic, individuals with T2DM present increased nocturnal urinary frequency, blurry vision, sores that heal slowly, tingling hands, and changes in mental status, among others [4].

Globally, around 500 million adults aged between 20 and 80 years were living with diabetes, according to a 2023 survey by the World Health Organization [5]. As of 2021, this represented 10.5% of the world's population in the age group. In Africa, an estimated 24 million people have diabetes, with an anticipated growth of 55 million by 2045. This is compounded by the fact that more than half (54%) of people living with diabetes in the African region are undiagnosed. In context with the report, 95% of persons diagnosed with diabetes had Type 2 diabetes [5]. In industrialized regions such as North America and Europe, prevalence is highest among adults over 65 years, driven largely by obesity, sedentary lifestyles, and aging populations. By contrast, developing regions experience an earlier onset of T2DM, often before the age of 45, associated with rapid urbanization, nutrition transitions, and limited healthcare access [5].

To counteract this health threat, many studies have documented the use of plants for management of T2DM [6–8]. There has been exploration of phytochemicals and their antidiabetic properties for commonly used medicinal plants such as *Allium cepa*, *Persea americana*, and *Acalypha wilkesiana* [8]. *A. wilkesiana* has been used in ethnobotanical medicine for diabetes management, but its pharmacological potential remains insufficiently characterized. The shrub, widely cultivated as an ornamental plant, is distinguished by its colorful foliage and is also traditionally applied in the treatment of skin infections, hypertension, and gastrointestinal disorders [9]. Phytochemical screening has identified flavonoids, tannins, saponins, terpenoids, and phenolic compounds, many of which are associated with hypoglycemic or antioxidant activities in other medicinal plants [10]. Unlike *Allium cepa* or *Persea americana*, which are well documented, *A. wilkesiana* represents an understudied but promising candidate for antidiabetic research. Its unique phytochemical profile and traditional relevance provide a compelling rationale for review.

This paper is aimed at thoroughly reviewing the current understanding of critical phytochemicals, pharmacological action, potency, and safety of *A. wilkesiana* in managing T2DM. This will enhance its validity in contemporary ethnobotanical medicine and provide insights into the urgent future research necessary to advance care among patients with T2DM.

## 2. Materials and Methods

A narrative review was thoroughly conducted by searching Google Scholar and PubMed databases. The keywords “Type 2 diabetes”, “Type 2 diabetes mellitus”, “t2dm”, “Acalypha wilkesiana”, “copper leaf”, “alpha-glucosidase inhibitors”, “glycemic control”, “blood sugar control”, “mechanism of action”, and “safety” were combined using the Boolean operators “AND” and “OR” to retrieve peer-reviewed papers published up to 2025. The retrieved articles were screened and analyzed based on their relevance to the review's objectives. Snowballing of the retrieved articles was then conducted to collate adequate data. Articles with information irrelevant to the review's aim were excluded. While preparing the final report of this review, all included articles were cross-checked. A total of 44 articles was considered in this review.

## 3. Results and Discussion

### 3.1. Current Treatment of T2DM

The management of T2DM involves lifestyle modifications, such as diet, exercise, and weight control. It also includes pharmacological interventions, where patients take medications such as oral antidiabetic drugs (OADs) and insulin injections [11]. Lifestyle changes, such as dietary adjustments, regular physical activity, and weight management, form the cornerstone of T2DM management [12]. The OADs are the first-line therapy for most patients with T2DM, and they can be classified into different categories based on their mechanisms of action [13]. The biguanide class includes medications like metformin, which reduces hepatic glucose synthesis and enhances peripheral glucose uptake [14]. This class is associated with side effects such as gastrointestinal disturbances, lactic acidosis, and vitamin B12 deficiency. Other classes of drugs include sulfonylureas and meglitinides which stimulate insulin secretion from pancreatic beta-cells [15]. Besides, they are accountable for side effects such as weight gain and hypoglycemia. It is therefore significant to monitor blood sugars when these medications are being administered.

Additionally, thiazolidinediones, such as pioglitazone and rosiglitazone, improve insulin sensitivity in muscle and adipose tissue [16]. Dipeptidyl peptidase-4 (DPP-4) inhibitors and glucagon-like peptide-1 (GLP-1) receptor agonists, such as sitagliptin and liraglutide, boost incretin-mediated insulin secretion while suppressing glucagon release [17]. The major side effects reported by patients who use these drugs include upper respiratory tract infections, urinary tract infections, hypersensitivity, gastrointestinal disturbances, hypoglycemia, pancreatitis, and thyroid cancer [17]. Sodium-glucose cotransporter-2 (SGLT-2) inhibitors, such as dapagliflozin and empagliflozin, inhibit renal glucose reabsorption and increase urinary glucose excretion [18]. This class has been reported to increase cases of genital infections, urinary tract infections, dehydration, ketoacidosis, and bone fractures [18] (see Table 1).

The choice of OADs depends on various factors, such as efficacy, safety, tolerability, cost, and patient preference [18]. The conventional OADs outlined in Table 1 are effective, but they have several significant drawbacks. Several important issues have been identified, including progressive loss of efficacy (secondary failure), weight gain (e.g., sulfonylureas and thiazolidinediones), hypoglycemia risks, gastrointestinal intolerance (e.g., metformin), and cardiovascular safety concerns such as risks of heart failure due to rosiglitazone or saxagliptin [19]. In addition, drug interactions with alcohol, anticoagulants, and antibiotics, among other drugs raise a concern. Genetic variability, such as polymorphisms in drug-metabolizing enzymes, transporters, and receptors can also affect drug response and toxicity [20]. Moreover, compliance issues such as poor adherence, discontinuation, and switching of therapy play a significant role in limiting proper treatment of T2DM disease. Ongoing studies in the field of healthcare are primarily concerned with the search for better treatment options for many noncommunicable diseases, including T2DM. According to Home and Mehta, [21] nanotechnology has rapidly expanded in several research domains, including phytomedicine to alleviate oxidative stress in diabetes [22]. Although OADs are satisfactory for most patients, insulin treatment continues to play a role in patients with definite T2DM or poor control of glycemia [23]. Insulin treatment varies from basal insulin to intensive therapy using rapid-acting analogues. Combination therapy in the form of metformin with SGLT-2 inhibitors or GLP-1 receptor agonists is now also a component of current strategies for overcoming drug resistance and enhancing outcomes [24].

However, the persistent limitations of conventional treatment have highlighted the significance of medicinal plants with antidiabetic properties. *A. wilkesiana* has exhibited significant hypoglycemic, antioxidant, and antihyperglycemic properties in preclinical settings, with efficacy comparable to conventional medications such as metformin and acarbose, as detailed in Tables 2 and 3. The findings indicate that *A. wilkesiana* could improve certain deficiencies of existing OADs while providing supplementary systemic advantages. Clinical trials remain necessary for confirming the safety and efficacy of this treatment in humans.

### 3.2. Overview of *A. wilkesiana*

*A. wilkesiana* belongs to the Euphorbiaceae plant family, one of the largest and most genetically diverse plant families that have been discovered for their significant role in the treatment and management of T2DM. It has nearly 322 genera and 8910 species ranging from large woody trees to weeds [32]. With such diversity, the family exhibits a wide array of phytoconstituents. Among the *Acalypha* species studied for their potential antidiabetic properties, *A. wilkesiana* has received considerable attention, likely due to its widespread distribution in tropical and subtropical regions and its documented medicinal applications [16]. *A. wilkesiana* also known by various local names such as copperleaf, fire-dragon, and Jacob's fruit, is not only valued for its medicinal properties but also widely used as an ornamental plant [15].

Medicinal significance of *A. wilkesiana* has been appreciated and recognized by different studies. In a research carried out to investigate the antifungal activity of this plant, Sherifat et al. observed antifungal activity of *A. wilkesiana* against *Trichophyton rubrum* and *Candida albicans*. Furthermore, they highlighted the plant's antimicrobial, anthelminthic, anticarcinogenic, anti-inflammatory, antioxidant, antimalarial, and hepatoprotective properties [15, 33]. The plant has been used in different areas of the world for a wide range of conditions such as antitumor in Nigeria, anti-inflammatory and treatment of headache and flu in Malaysia, regulation of menstrual flow in Fiji, and for management of rheumatic pains and swellings in Central America [34]. Additionally, an ethnobotanical study of *A. wilkesiana* revealed high efficacy in the treatment of arterial hypertension in Oyem, Northern Gabon. These results form a database for phytochemical studies for new antihypertensive compounds that can be used concurrently in the treatment of hypertensive diabetic patients [9].

### 3.3. Extraction and Phytochemicals of *A. wilkesiana*

Studies looking at *A. wilkesiana* have generally focused on three main parts of the plant: the root bark, the stem bark, and the leaves. Each of these carries a mix of useful compounds such as saponins, flavonoids, alkaloids, terpenoids, and tannins, but their reported strengths are not the same. Root bark extracts are often highlighted for lowering postprandial glucose, stem bark for its strong antioxidant profile, while the leaves have attracted the most attention overall because of their broader range of effects, such as improving insulin sensitivity and helping preserve *β*-cell function [35].

**Figure 1:**
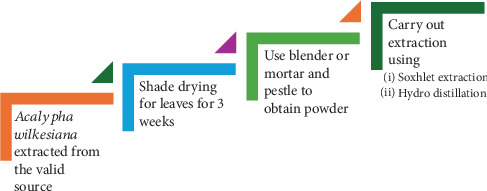
The isolation process undertaken to obtain a pure extract of *Acalypha wilkesiana*.

To obtain these compounds, researchers have tried different solvents like water, ethanol, methanol, and ethyl acetate, depending on the target isolate [16]. Among these, ethanol and water are most often used, and the bulk of published studies concentrate on leaf extractions [36, 37]. These methods described are similar: leaves are typically dried, powdered, and then processed through techniques such as Soxhlet extraction or hydrodistillation to produce crude extracts or essential oils. A general outline of these approaches is illustrated in Figure 1 [37, 38].

Upon analysis of these extracts, scientists consistently reported a rich chemical profile. The leaves contain polyphenols, terpenoids, flavonoids, and glycosides, along with tannins, alkaloids, and steroids. Some studies note that ethanol extractions reveal a broad spectrum of these compounds, while water extractions often bring out higher levels of saponins [18, 39]. These groups of compounds are not just chemical curiosities; they are associated with diverse biological activities, from antioxidant and antiviral effects to cardiovascular and antimicrobial benefits [39].

**Figure 2:**
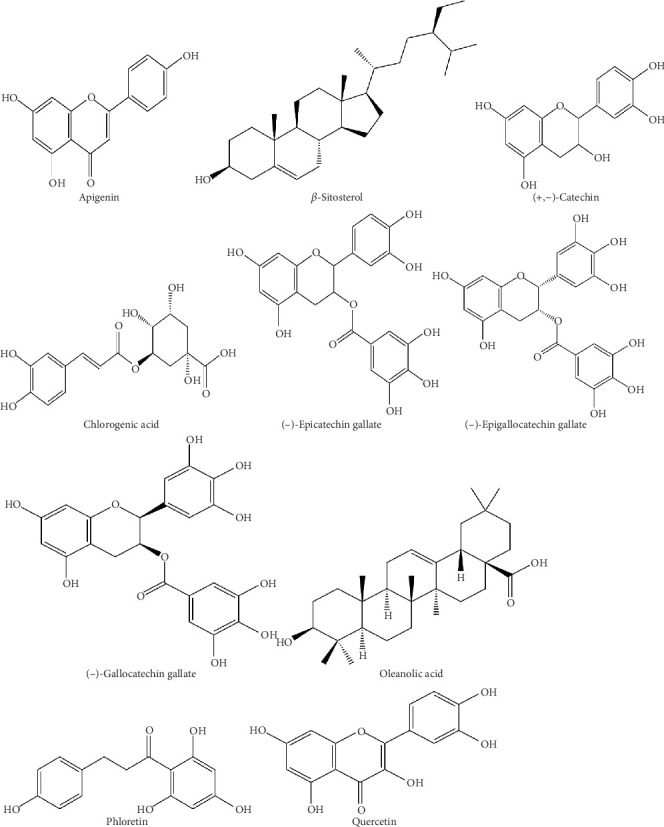
Chemical structures of bioactive elements found in *Acalypha wilkesiana* [16, 18, 39].

Several individual compounds stand out for their relevance to diabetes. For example, saponins like aescin and oleanolic acid have been linked with *α*-glucosidase inhibition and reduced glucose absorption [27]. Flavonoids such as quercetin and kaempferol act in insulin-like ways, enhancing glucose uptake while also protecting against oxidative stress [16, 35]. Phenolic acids like chlorogenic acid appear to interfere with enzymes such as *α*-amylase and PTP1B, supporting better insulin signaling [25]. Other receptors on methanolic extracts describe compounds such as rutin, ferulic acid, caffeic acid, and luteolin, which add further antidiabetic and lipid-modulating effects [26]. Representative chemical structures of these phytochemicals are provided in Figure 2, while a concise summary of their classes, properties, and biological targets is presented in Table 2.

### 3.4. Mechanism of Action, Safety, Potency, and Efficacy of *A. wilkesiana*

The antidiabetic potential of *A. wilkesiana* has been consistently supported by various in vivo studies, which highlight multiple mechanisms of action. A central pathway involves the inhibition of carbohydrate-digesting enzymes, particularly *α*-amylase and *α*-glucosidase, thereby delaying glucose absorption and reducing postprandial spikes [16, 40]. For example, Iyamu et al. demonstrated that ethanolic root bark extracts inhibited *α*-glucosidase at levels (50.48%–56.14%) comparable to the standard drug acarbose (51.12%–58.01%), suggesting a pharmacological similarity that warrants further exploration [39]. Complementary findings have shown that different plant parts yield varying inhibitory potencies, with root bark ethanolic extracts displaying the lowest IC₅₀ values against both *α*-glucosidase and *α*-amylase, indicating the highest hypoglycemic potential. However, this potency also raises concerns about toxicity, as the narrow therapeutic window could limit safe application.

Beyond enzyme inhibition, *A. wilkesiana* appears to enhance glucose utilization directly by stimulating uptake in muscle and adipose tissues, while also improving insulin sensitivity [42]. Importantly, it protects pancreatic *β*-cells by enhancing insulin secretion and shielding them from oxidative stress-induced apoptosis. Such effects are linked to its antioxidant constituents, particularly geraniin and corilagin, which exhibit vigorous free-radical scavenging activity [31]. These dual actions supporting *β*-cell survival and reducing oxidative stress address key pathophysiological factors in T2DM. Additional animal studies further strengthen this evidence [43].

Isirima and Uahomo also attributed the mechanism of action of *A. wilkesiana* to glibenclamide, a secretagogue that binds sulfonylurea receptors and stimulates insulin release from the pancreas. Their study also described that *A. wilkesiana* has a protective function on the liver and spleen. This was supported by the ability of the plant to reverse alloxan-induced insult on the liver and spleen of rat models. They further concluded the antidiabetic potential of *A. wilkesiana* when they exposed it to a diabetes-induced rat [40].

With regards to safety, Forcados et al. reported a dose-dependent increase in major liver markers, serum aspartate aminotransferase (AST), alkaline phosphatase (ALP), and alanine aminotransferase (ALT) when *A. wilkesiana* was administered to albino rats. Conversely, when the rats were administered a dose of 100 mg/kg, a decrease in the levels of the major liver markers was noticed. Hence, 100 mg/kg doses and below portrayed hepatoprotection while higher doses showed otherwise [42]. Moreover, the leaves of *A. wilkesiana* have been reported to have no deleterious effects on the morphology of red blood cells of diabetic rabbits [41]. Additionally, they demonstrated the ability to restore damaged cells of the pancreas and spleen caused by alloxan-induced diabetes [44]. This property of the plant provides a greater advantage over synthetic drugs of T2D as it does not only lower blood glucose levels but also ameliorates the functions of critical organs. Unlike synthetic drugs, which do indeed lower glucose, they cause side effects, harming the vital organs.

From the research conducted by Isirima and Uahomo, *A. wilkesiana* was efficacious in reducing hyperglycemia in experimental animals in a dose-dependent manner when they used 200 and 400 mg/kg of the plant extract [40]. Another study by Iyamu et al. also supported the hypoglycemic activity of the plant via *α*-glucosidase inhibition [39]. Furthermore, a study on the inhibitory activity of *A. wilkesiana* extracts against *α*-amylase and *α*-glucosidase, ethanolic root bark, and stem bark extracts demonstrated the lowest minimal extract concentration required for 50% enzyme inhibition (IC_50_) as compared to ethyl acetate and aqueous extracts. Stem ethanolic extracts had IC_50_ (*μ*g mL^–1^) values of −37.10 ± 2.71^a^ and >1000^g^ towards *α*-glucosidase and *α*-amylase, respectively. Besides, the IC_50_ (*μ*g mL^–1^) values for the root bark ethanolic extracts against *α*-glucosidase and *α*-amylase were 35.75 ± 1.95^a^ and 6.25 ± 1.05^a^, respectively [16]. Among the two parts of the plant, the lowest values of IC_50_ were for the root bark extract, which implies the extract concentration required to produce half of the maximum therapeutic effect. Moreover, the results suggest that root bark ethanolic extract is a potential source of phytochemicals with the highest hypoglycemic potency, and this promises to serve as a direction for better extraction process (see Table 3).

Overall, these findings position *A. wilkesiana* as a promising candidate for antidiabetic therapy, with mechanisms spanning enzyme inhibition, insulin sensitization, antioxidant defense, and organ protection. However, the balance between potency and toxicity, especially concerning extracts with very low IC₅₀ values remains a critical gap for future research.

The antidiabetic efficacy of *A. wilkesiana* has been evidenced in streptozotocin-induced animal models, whereas administration of leaf extracts significantly reduced hyperglycemia [43]. In a study, diabetic mice treated with streptozotocin alone exhibited a significant 445.2% increase in serum glucose after 30 days, while cotreatment with *A. wilkesiana* leaf extract restricted this rise to 305.6%, indicating a 139.6% disparity between the groups [43]. This significant decrease highlights the extract's therapeutic efficacy in the management of T2DM.

Therefore, it is crucial to determine the dosage of the plant extract that produces optimal hyperglycemic activity in humans [18]. In diabetic rats, a methanolic leaf extract at 40 mg/100 g body weight resulted in a 77.37% decrease in blood glucose, closely paralleling the 76.50% decrease reported with metformin [18]. Alongside managing glucose levels, the extract improved liver and kidney function indicators, lipid profiles, and hematological markers, suggesting broader systemic benefits that might enhance treatments.

## 4. Limitations

This review considered research studies performed using animal models and may not fully replicate the human condition of T2DM. Thus, the findings have limited applicability to human treatment. Moreover, the absence of clinical trials in humans indicates inadequate evidence concerning the safety, efficacy, and appropriate dosage of *A. wilkesiana* extracts for human application. The review also lacks explicit guidance for dosage and duration, and the observed toxicity at higher concentrations in rats indicates a necessity for future investigation into safe usage. Additionally, this review missed the potential variability in extract efficacy due to factors such as plant age or extraction process. Therefore, while the findings are promising, further research, particularly human clinical trials, is essential to confirm the therapeutic potential of this plant.

## 5. Conclusion

This comprehensive review has revealed that extracts of *A. wilkesiana* can lower blood glucose by modulating key enzymes involved in glucose metabolism and insulin signaling. They inhibit *α*-glucosidase and *α*-amylase enzymes, which are usually responsible for the breakdown of carbohydrates into glucose. It has been observed that ethanolic root bark extracts of the plant are more potent against the enzymes because of their lowest demonstrated IC_50_ value among other solvents and the standard acarbose, which works in the same biological pathway. Additionally, it can reverse the dysfunction of critical organs such as the spleen and pancreas in alloxan-induced models. Exploring the mechanisms of action, potency, and safety of *A. wilkesiana* is paramount to validate its ethnobotanical use. It also promises advances in developing antihyperglycemic agents that are more efficacious and safer than synthetic agents. Nevertheless, the safety profile of this plant is still an area of concern because there are no human-tailored standard doses. Thus, further research should focus on clinical trials to establish standard doses and ensure the safety profile of *A. wilkesiana* in T2DM management.

## Figures and Tables

**Table 1:** Categories of oral antidiabetic drugs (OADs), examples, mechanism of action, and side effects.

**Drug class**	**Examples**	**Mechanism of action**	**Side effects**	**References**
Biguanides	Metformin	Reduce hepatic glucose production and improve peripheral glucose absorption.	Gastrointestinal disturbances	[14]
Sulfonylureas	Glyburide (glibenclamide) Glipizide	Stimulate secretion of insulin from pancreatic beta cells	Hypoglycemia, weight gain, allergic reactions	[15]
Meglitinides	Repaglinide Nateglinide	Increase insulin release from pancreatic beta cells in a glucose-dependent way.	Hypoglycemia, weight gain	[15]
DPP-4 inhibitors	Sitagliptin liraglutide	Inhibit the breakdown of incretin hormones (GLP-1 and GIP), which boost insulin secretion and inhibit glucagon secretion.	Upper respiratory tract infections, urinary tract infections, pancreatitis, hypersensitivity	[17]
GLP1R agonists	Exenatide Liraglutide	Imitate the activity of GLP-1 by increasing insulin secretion, suppressing glucagon secretion, prolonging stomach emptying, and decreasing appetite.	Gastrointestinal disturbances, hypoglycemia, pancreatitis, thyroid cancer	[17]
SGLT-2 inhibitors	Dapagliflozin empagliflozin	Inhibit the reabsorption of glucose in the renal tubules.	Genital infections, urinary tract infections, dehydration, ketoacidosis, bone fractures	[18]

**Table 2:** A summary of properties and targets for some bioactive elements found in *Acalypha wilkesiana.*

**Name**	**Class**	**Properties**	**Specific target**	**References**
Catechin	Flavan-3-ol	Antioxidant	*α*-Glucosidase	[15]
Apigenin	Flavone	Anti-inflammatory	*α*-Amylase	[15, 25]
*β*-Sitosterol	Phytosterol	Cholesterol-lowering	Lipase	[26]
Phloretin	Flavonoid	Antioxidant	*α*-Glucosidase	[25, 27]
Chlorogenic acid	Phenolic acid	Antioxidant, enzyme inhibitory	*α*-Amylase, protein tyrosine phosphatase 1B (PTP1B)	[25]
Quercetin	Flavonoid	Antioxidant, insulin-like	*α*-Amylase *α*-Glucosidase	[26, 27]
Kaempferol (kaempferol 3-O-rutinoside)	Flavonoid/flavonol glycoside)	Antioxidant	*α*-Glucosidase	[27]
Aescin	Saponin	Anti-inflammatory, antioxidant	*α*-Glucosidase *α*-Amylase	[15, 27]
Oleanolic acid	Saponin	Antidiabetes, anti-inflammatory, antioxidant, anti-HIV, antimutagenic	*α*-Glucosidase Pancreatic and salivary *α*-amilase	[15, 25, 28]
Geraniin Gallic acid Corilagin	Tannins	Antihyperglycemic (increase insulin sensitivity), antihypertensive, antioxidant, anticancer, anti-infective, antiviral	*α*-Glucosidase, *α*-amylase Protein kinase B (Akt)	[29, 30]

**Table 3:** Potency (IC_50_) values of different *A. wilkesiana* extracts and standard acarbose against alpha-glucosidase and alpha-amylase [16, 31].

**Extract**	**IC **_**50**_ ** (*μ*g/mL)**	
	***α*-Glucose**	***α*-Amylase**
Root bark		
Aqueous	127.25 ± 23.60^d^	151.04 ± 12.59^e^
Ethanol	35.75 ± 1.95^a^	6.25 ± 1.05^a^
Ethyl acetate	124.04 ± 16.72^d^	98.65 ± 15.11^d^
Stem bark		
Aqueous	267.94 ± 24.90^e^	>1000^g^
Ethanol	37.10 ± 2.71^a^	> 1000^g^
Ethyl acetate	242.99 ± 41.83^e^	274.55 ± 31.09^f^
Leaves		
Aqueous	88.35 ± 15.37^c^	118.9 ± 19.3^d,e^
Ethanol	67.18 ± 7.65^b^	75.35 ± 8.25^c^
Ethyl acetate	74.63 ± 2.87^b^	296.18 ± 37.18^f^
Standard (acarbose)	36.27 ± 1.84	53.77 ± 3.95

*Note:* Data are presented as mean ± SD values for triplicate determinations. IC_50_ is the minimal extract concentration required for 50% inhibition. Different superscripts (a–g) in a column for a given parameter indicate a significant difference (*p* < 0.05).

## Data Availability

The data used to support the findings of this study are included within the article.

## Nomenclature

T2DM: Type 2 diabetes mellitus
IC_50_: minimum extract concentration required for 50% inhibition
OADs: oral antidiabetic drugs

## References

[1] Rasouli H., Ramalho T. C., Popović-Djordjević J. B., Devkota H. P. (2023). Editorial: New Opportunities in Drug Design for the management and Treatment of Type 2 Diabetes. *Frontiers in Pharmacology*.

[2] Reed J., Bain S., Kanamarlapudi V. (2021). A Review of Current Trends With Type 2 Diabetes Epidemiology, Aetiology, Pathogenesis, Treatments and Future Perspectives. *Diabetes, Metabolic Syndrome and Obesity*.

[3] Młynarska E., Czarnik W., Dzieża N. (2025). Type 2 Diabetes Mellitus: New Pathogenetic Mechanisms, Treatment and the Most Important Complications. *International Journal of Molecular Sciences*.

[4] CDC Diabetes (2024). Symptoms of Diabetes. https://www.cdc.gov/diabetes/signs-symptoms/index.html.

[5] Analytical Fact Sheet: Diabetes, a Silent Killer in Africa. https://medbox.org/document/analytical-fact-sheet-diabetes-a-silent-killer-in-africa.

[6] Jacob B., Narendhirakannan R. T. (2019). Role of Medicinal Plants in the Management of Diabetes Mellitus: A Review. *3 Biotech*.

[7] Tienda-Vázquez M. A., Melchor-Martínez E. M., Elizondo-Luévano J. H. (2023). Antidiabetic Plants for the Treatment of Type 2 Diabetes Mellitus and Associated Bacterial Infections. *Processes*.

[8] Muema F. W., Nanjala C., Oulo M. A., Wangchuk P. (2023). Phytochemical Content and Antidiabetic Properties of Most Commonly Used Antidiabetic Medicinal Plants of Kenya. *Molecules*.

[9] Omage K., Azeke M. A., Omage S. O. (2018). Evaluation of the Efficacy of *Acalypha wilkesiana* Leaves in Managing Cardiovascular Disease Risk Factors in Rabbits Exposed to Salt-Loaded Diets. *Clinical Phytoscience*.

[10] Labu Z. K., Karim S., Arifuzzaman S. (2025). The South Asian *Acalypha* Species: A Comprehensive Review on Traditional Uses, Phytochemistry and Pharmacological Aspect. *Chemistry & Biodiversity*.

[11] Nasykhova Y. A., Tonyan Z. N., Mikhailova A. A., Danilova M. M., Glotov A. S. (2020). Pharmacogenetics of Type 2 Diabetes—Progress and Prospects. *International Journal of Molecular Sciences*.

[12] American College of Cardiology/American Heart Association Task Force on Practice Guidelines, Obesity Expert Panel (2014). Expert Panel Report: Guidelines (2013) for the Management of Overweight and Obesity in Adults. *Obesity*.

[13] Heo C. U., Choi C. I. (2019). Current Progress in Pharmacogenetics of Second-Line Antidiabetic Medications: Towards Precision Medicine for Type 2 Diabetes. *Journal of Clinical Medicine*.

[14] Nie T., Cooper G. J. S. (2021). Mechanisms Underlying the Antidiabetic Activities of Polyphenolic Compounds: A Review. *Frontiers in Pharmacology*.

[15] Odoh U. E., Ndubuokwu R. I., Inya-Agha S. I., Osadebe P. O., Uzor P. F., Ezejiofor M. (2014). Antidiabetic Activity and Phytochemical Screening of *Acalypha wilkesiana* (Euphorbiaceae) Mull Arg. Roots in Alloxan-Induced Diabetic Rats. *Scientific Research and Essays*.

[16] Oyebode O., Erukainure O. L., Koorbanally N. A., Islam S. (2025). *Acalypha wilkesiana* ‘Java White’: Identification of Some Bioactive Compounds by Gc-Ms and Their Effects on Key Enzymes Linked to Type 2 Diabete. https://www.researchgate.net/publication/327867783_Acalypha_Wilkesiana_'Java_White'_Identification_of_Some_Bioactive_Compounds_by_Gc-Ms_and_Their_Effects_on_Key_Enzymes_Linked_to_Type_2_Diabete.

[17] Oluduro A. O., Bakare M. K., Omoboye O. O., Dada C. A., Olatunji C. I. (2011). Antibacterial Effect of Extracts of *Acalypha wilkesiana* on Gastrointestinal Tract Pathogens and Bacteria Causing Skin Infections in Neonates. https://scispace.com/pdf/antibacterial-effect-of-extracts-of-acalypha-wilkesiana-on-4m9sknyldj.pdf.

[18] El-Khateeb A. Y., Azzaz N. A. K. E., Mahmoud H. I. (2014). Phytochemical Constituents, Hypoglycemic and Haematological Effects of Methanolic *Acalypha wilkesiana* Leaves Extract on Streptozotocin-Induced Diabetic Rats. *European Journal of Chemistry*.

[19] ElSayed N. A., Aleppo G., Aroda V. R. (2023). 9. Pharmacologic Approaches to Glycemic Treatment: Standards of Care in Diabetes—2023. *Diabetes Care*.

[20] Inshutiyimana S., Ramadan N., Razzak R. A., Al Maaz Z., Wojtara M., Uwishema O. (2024). Pharmacogenomics Revolutionizing Cardiovascular Therapeutics: A Narrative Review. *Health Science Reports*.

[21] Home P. D., Mehta R. (2021). Insulin Therapy Development Beyond 100 Years. *The Lancet Diabetes & Endocrinology*.

[22] Alfarisi H., Wresdiyati T., Sadiah S., Juliandi B. (2022). Nanoextract of Acalypha hispida Leaves Increases Antioxidant Defense and Suppresses Microstructure Damage in Liver and Kidney of Diabetic Rats. *Journal of Applied Pharmaceutical Science*.

[23] van Baar M. J. B., van Ruiten C. C., Muskiet M. H. A., van Bloemendaal L., IJzerman R. G., van Raalte D. H. (2018). SGLT2 Inhibitors in Combination Therapy: From Mechanisms to Clinical Considerations in Type 2 Diabetes Management. *Diabetes Care*.

[24] Kuete V., Kuete V. (2014). 22- Physical, Hematological, and Histopathological Signs of Toxicity Induced by African Medicinal Plants. *Toxicological Survey of African Medicinal Plants [Internet]*.

[25] Malaguti M., Angeloni C., Hrelia S. (2013). Polyphenols in Exercise Performance and Prevention of Exercise-Induced Muscle Damage. *Oxidative Medicine and Cellular Longevity*.

[26] Tresina P. S. (2022). Oleanolic Acid - an Overview|ScienceDirect Topics. https://www.sciencedirect.com/topics/chemistry/oleanolic-acid.

[27] Cvetanović A. (2021). Apigenin - an Overview|ScienceDirect Topics. https://www.sciencedirect.com/topics/agricultural-and-biological-sciences/apigenin.

[28] Castellano J. M., Ramos-Romero S., Perona J. S. (2022). Oleanolic Acid: Extraction, Characterization and Biological Activity. *Nutrients*.

[29] Anokwuru C. P., Sinisi A., Samie A., Taglialatela-Scafati O. (2015). Antibacterial and Antioxidant Constituents of *Acalypha wilkesiana*. *Natural Product Research*.

[30] Variya B. C., Bakrania A. K., Patel S. S. (2020). Antidiabetic Potential of Gallic Acid From *Emblica officinalis*: Improved Glucose Transporters and Insulin Sensitivity Through PPAR-*γ* and Akt Signaling. *Phytomedicine*.

[31] Didunyemi O. (2023). Comparative *α*-Amylase and *α*-Glucosidase Inhibitory Potency and Mode of Inhibition of Aqueous Leaf Extracts of *Acalypha wilkesiana* ‘Green’ and *Acalypha wilkesiana*‘Red’. *Journal of Biomedical Sciences*.

[32] Sherifat K. O., Itohan A. M., Adeola S. O., Adeola K. M., Aderemi O. L. (2022). Anti-Fungal Activity of *Acalypha wilkesiana*: A Preliminary Study of Fungal Isolates of Clinical Significance. *African Journal of Infectious Diseases*.

[33] Abiola O. A. (2020). Acalypha wilkesiana: Prospects as an Agricultural Biocide. *World Journal of Advanced Research and Reviews*.

[34] Mendame W. L. M., Mintsa B. A. E., Nguema A.-M. N., Pambo A. B. P., Ibrahim E. T. (2022). Ethnobotanical Study of *Acalypha wilkesiana* (Euphorbiaceae), a Plant Used in the Treatment of Arterial Hypertension in Oyem in Northern Gabon. *International Journal of Biomolecules and Biomedicine*.

[35] Kadiri H. E., Ossai H. U. (2023). Ameliorative Potential of *Acalypha wilkesiana* Leaf Extract (subsp. macrophylla) on Cyanide-Induced Renal Damaged Wister rats. *Scientific African*.

[36] Ify O. A., Ogbonna S. U. A., Ifeanyi U. E. (2021). Biochemical Analysis and Antimicrobial Activity of Ethanolic and Aqueous Leaf Extracts of *Acalypha wilkesiana* var *morea* Müll. Arg. (Copperleaf). *Journal of Botany Research*.

[37] Kingsley O., Marshall A. A. (2014). Medicinal Potential of Acalypha wilkesiana Leaves. *Advances in Research*.

[38] Oladunmoye M. K. (2006). Comparative Evaluation of Antimicrobial Activities and Phytochemical Screening of Two Varieties of Acalypha wilkesiana. https://scialert.net/abstract/?doi=tasr.2006.538.541.

[39] Iyamu A. O., Otamere H. O., Akpamu U. (2020). In Vitro *Α*-Glucosidase Inhibitory and In Vivo Glucose Digestion Activities of Ethanol Leaf Extract of Acalypha wilkesiana in Normoglycaemic Rats. *BJSTR*.

[40] Isirima J. C., Uahomo P. O. (2023). Effect of Acalypha wilkesiana on Oxidative Stress and Histopathology of Liver and Kidney in Alloxan-Induced Diabetic Albino Rats. *Journal of Complementary and Alternative Medical Research*.

[41] Ikewuchi J. C., Onyeike E. N., Uwakwe A. A., Ikewuchi C. C. (2011). Effect of Aqueous Extract of the Leaves of *Acalypha wilkesiana* ‘Godseffiana’ Muell Arg (Euphorbiaceae) on the Hematology, Plasma Biochemistry and Ocular Indices of Oxidative Stress in Alloxan Induced Diabetic Rats. *Journal of Ethnopharmacology*.

[42] Forcados G. E., Chinyere N., Shu M. L. (2025). Acalypha wilkesiana: Therapeutic and Toxic Potential. *Journal of Medical & Surgical Pathology*.

[43] Al-Attar A. M. (2010). Physiological Study on the Effect of *Acalypha wilkesiana* Leaves Extract on Streptozotocin-Induced Experimental Diabetes in Male Mice. *CRM*.

[44] Isirima J. C., Uahomo P. O. (2023). *Acalypha wilkesiana* Exhibits Antihyperglycemic Potentials and Ameliorates Damages to Pancreas and Spleen of Diabetic Rat Model. *Saudi Journal of Biomedical Research*.

